# Supplementary material for: Pluripotent stem cell-derived models of neurological diseases reveal early transcriptional heterogeneity
Source: Genome Biol. 2021 Mar 4;22:73. doi: 10.1186/s13059-021-02301-6 (PMC7934477; doi:10.1186/s13059-021-02301-6)
Supplement: Supplementary file 1 — Additional file 1: Supplementary Figures and Figure legends S1-S6 and Supplementary Methods. [file 13059_2021_2301_MOESM1_ESM.pdf]

Additional file 1: Supplemental Information for "Pluripotent stem cell derived models of neurological diseases reveal early transcriptional heterogeneity", Sorek et al. Supplementary Figures and Figure legends S1-S6 and Supplementary Methods.

Supplementary Figures and Figure legends

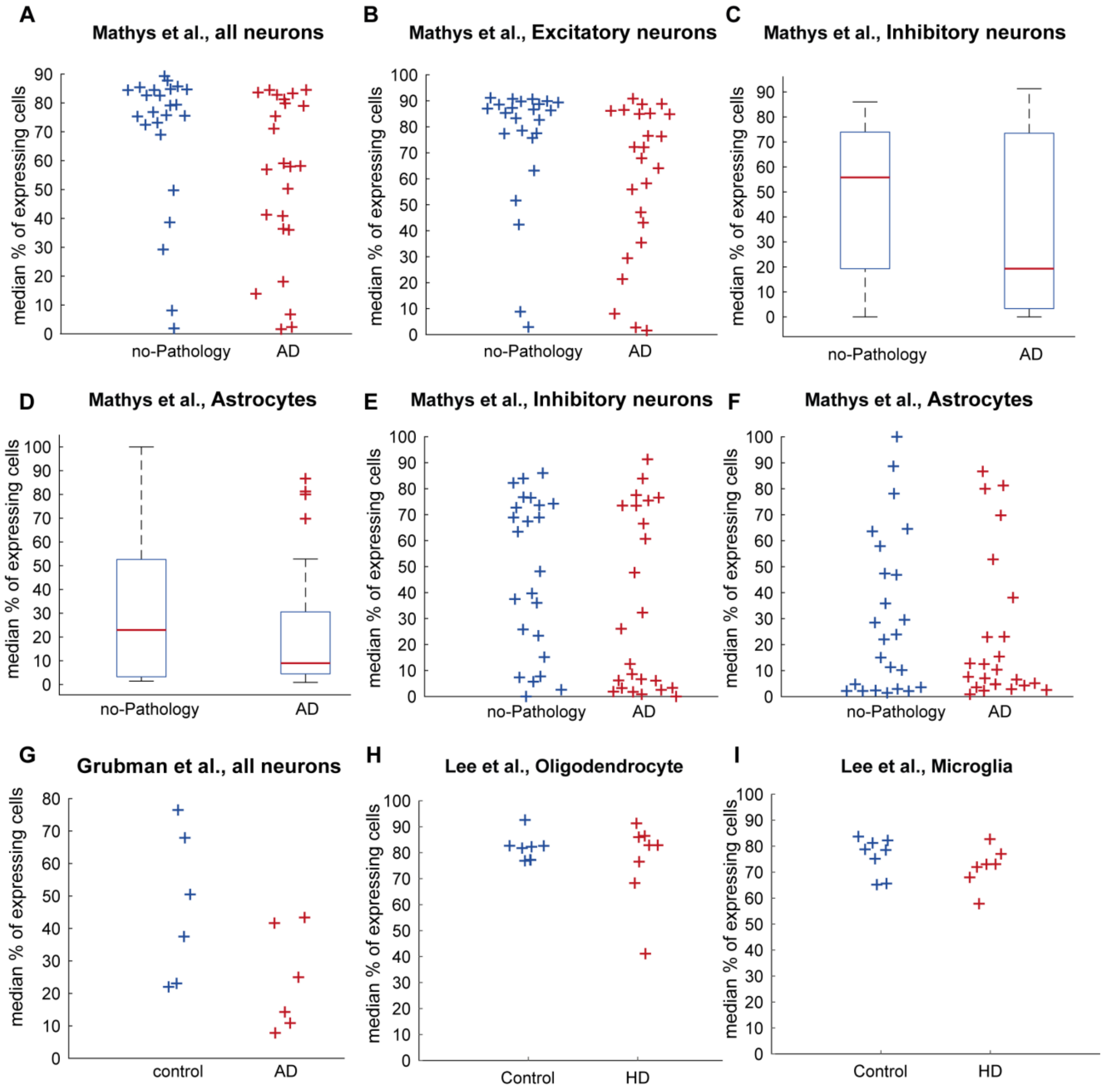

Figure S1. Transcriptional heterogeneity in AD adult neurons. Related to Figure 1. (**a-f**) The median percentage of expressing cells of the most 200 expressed genes for all adult neurons (**a**), excitatory neurons (**b**), inhibitory neurons (**c**) and astrocytes (**d**) in healthy and AD subjects from Mathys et al. and the corresponding boxplots for inhibitory neurons and astrocytes (**c** and **e**, respectively). **g** The median percentage of expressing cells of the most 100 expressed genes for all adult neurons in control and AD subjects from Grubman et al. **h-i** The median percentage of expressing cells of the most 200 expressed genes in healthy and HD subjects in Oligodendrocytes (**h**) and Microglia (**i**) from Lee et al.

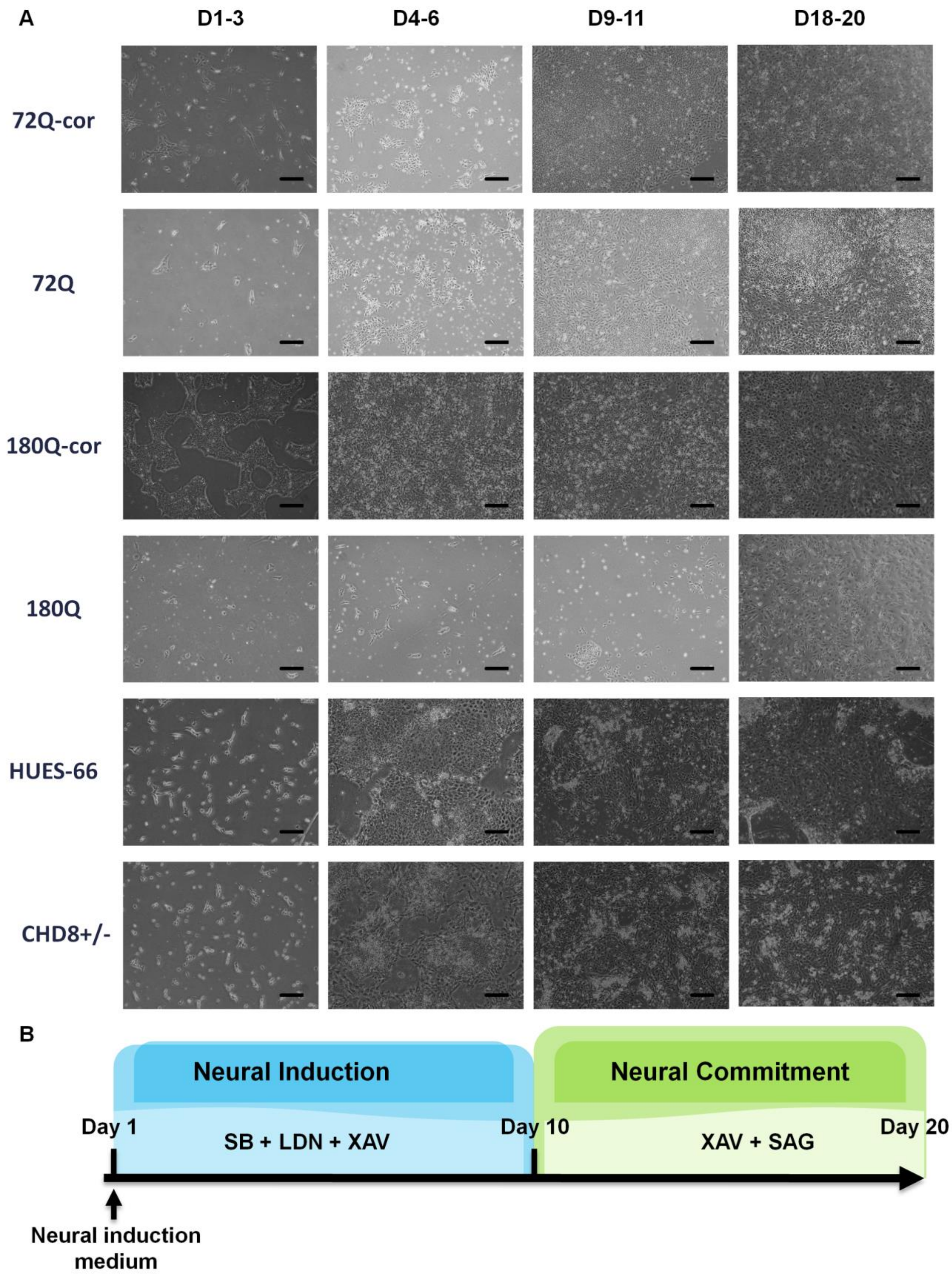
A
D1-3
D4-6
D9-11
D18-20
72Q-cor
72Q
180Q-cor
180Q
HUES-66
CHD8+/-
B
Neural Induction
Neural Commitment
Day 1
Day 10
Day 20
SB + LDN + XAV
XAV + SAG
Neural induction medium

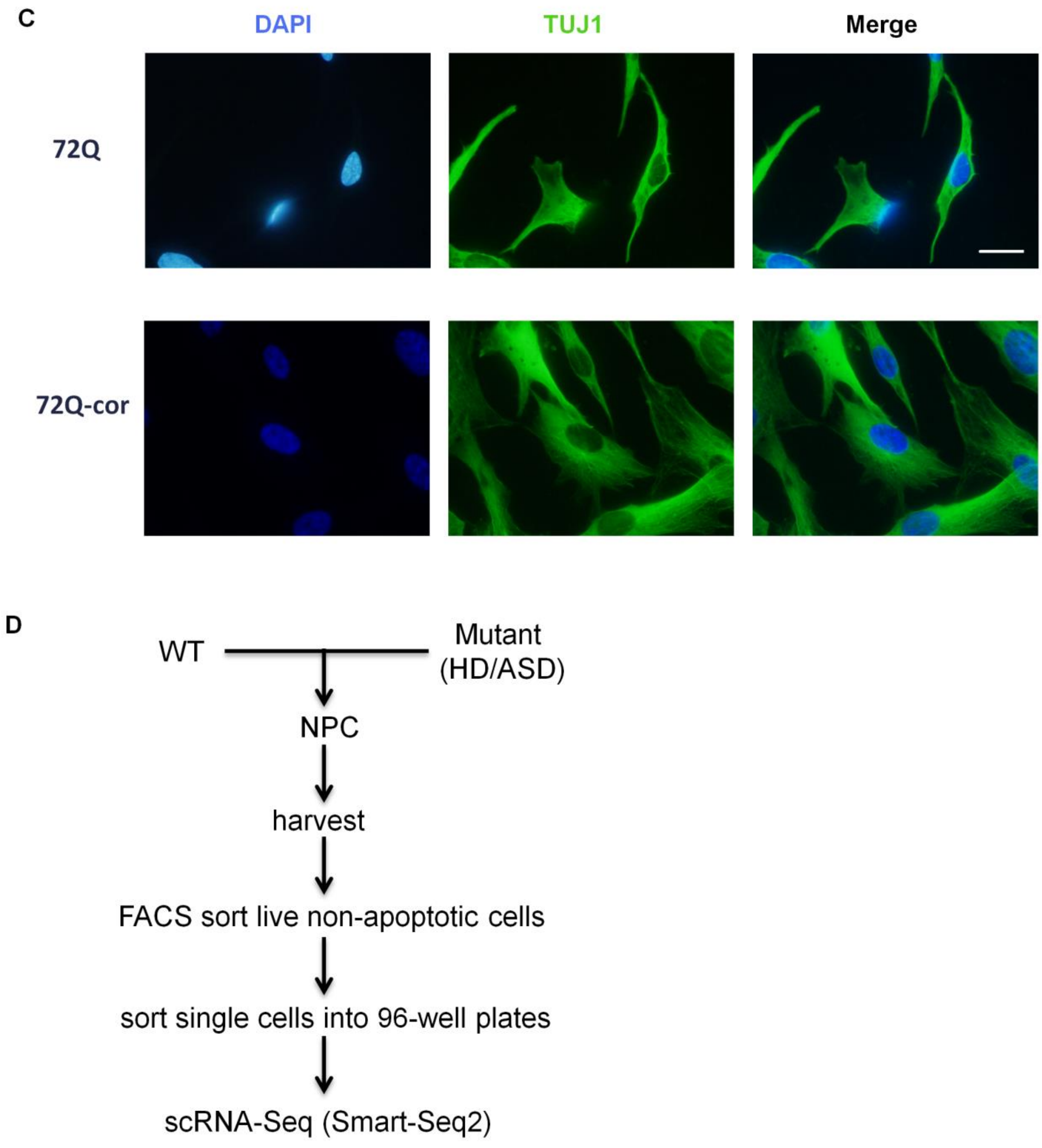

Figure S2. Experimental setup of scRNA-Seq of neuronal progenitor cells in culture. Related to Methods. a During differentiation, cells gradually acquire neuronal identity. Shown are days selected stages of the protocol. Scale bars = 50 μm. **b** Overview of the neuronal differentiation protocol of pluripotent stem cells. **c** NPCs express the neuronal marker TUJ1. Shown are example of the mutant and corrected 72Q cells. Scale bars = 10 μm **d** Experimental pipeline.

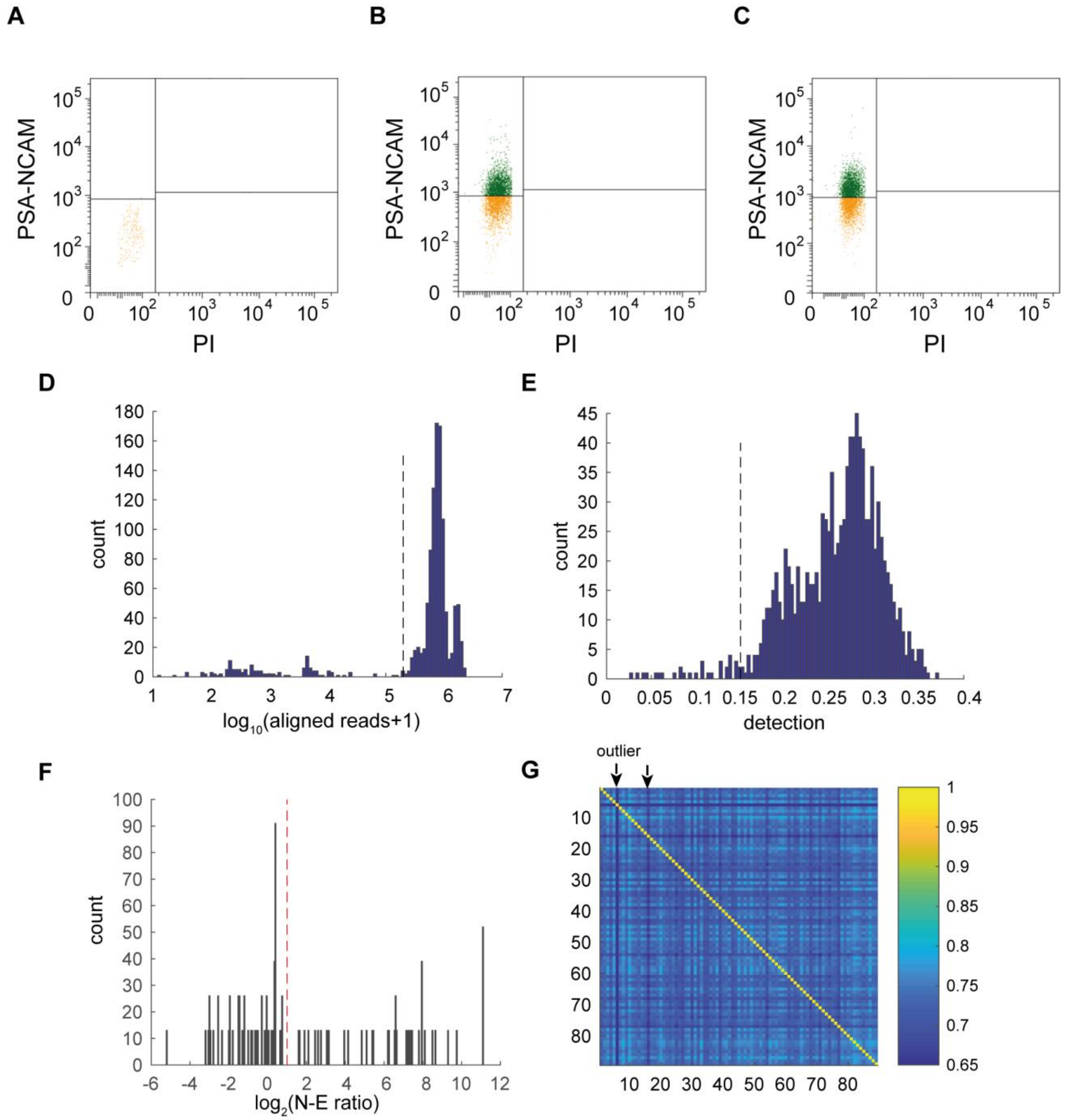

Figure S3. Experimental and computational selection of high-quality NPC for analysis. Related to Methods. a-c Cells are FACS-sorted using Annexin-V and PI to filter out apoptotic and dead cells, respectively, and to select for NPCs based on the expression of PSA-NCAM. Dark green represents cells which are positive for PSA-NCAM and negative for both Annexin-V (not shown) and PI. **a** hESC serve as negative controls for PSA-NCAM. **b-c** A large percentage of the mutant (here 72Q) and WT (72Q-corrected) NPCs, respectively, are intact and enriched for PSA-NCAM. **d** Histogram of total number of reads aligned to the transcriptome in all cells. **e** Histogram of percentage of detected genes in every cell. **f** Histogram of neural-to-embryonic **n-e** ratio of the cells (see Methods for definition). In **d-f** black dashed line represents lower threshold for filtering out cells. See Methods for more details. **g** Example of Spearman correlation between every 2 cells in the 72Q iPSC-derived NPCs. Arrows point at outliers.

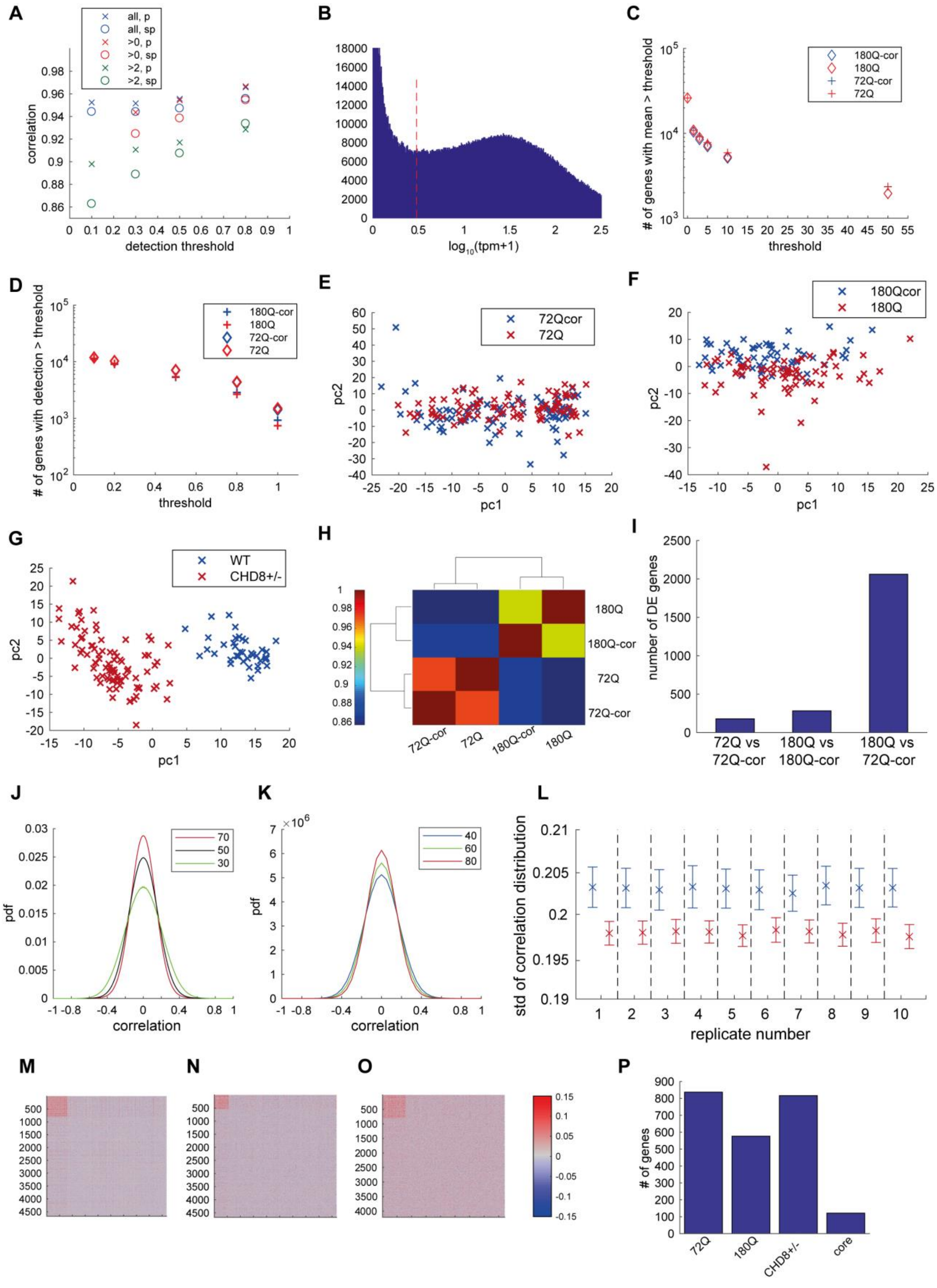

Figure S4. Correlation between single-cell and bulk data and HD transcriptional changes based on average expression level and pairwise gene correlations. Related to Figure 2. a Correlation of bulk data and average expression of single-cell data when including all values ("all"), only positive value (">0") and only reliable values (">2")

using threshold from **(b)**. "sp" stands for Spearman correlation and "p" stands for Pearson correlation. **b** Distribution of normalized expression levels in single-cell data. Red dashed line marks threshold used for detection. **c** Number of genes detected in single-cell data above different expression thresholds. **d** Number of genes detected in single-cell data above different thresholds of fraction of cells. **e-g** PCA of the 72Q, 180Q and CHD8$^{+/-}$ isogenic system, respectively. **h** Spearman correlation between mean expression in HD and WT cell lines. **i** Number of differentially expressed (DE-) genes in the HD isogenic systems and between the non-isogenic pair of 180Q mutant and the 72Q-corrected. **j** The correlation distribution depends on the number of cells; The larger the number of initial pool of cells, the smaller the correlations are in absolute value (see Methods). Shown are the distribution of pairwise gene correlations as a function of the number of cells used for the calculation out of the initial pool. The results shown are based on the average of 100 random samplings from the initial population of cells. **k** Given the same size of initial pool of cells, the correlation distribution depends on the number of cells drawn for the analysis; The larger the number of drawn cells, the smaller the correlations are in absolute value (see Methods). The results are based on summation of 50 random samples for each number of initial pool of cells. Results in (**j**) and (**k**) are shown for the 72Q-cor cell NPCs. **l** The stds of the correlation distributions in 10 replicates of random initial pools averaged over 1000 random selections of 30 cells in the 72Q isogenic system. Blue, WT; red, mutant. **m-o** Cluster analysis based on the difference of all pairwise correlations between WT and mutant cells in 72Q, 180Q and CHD8$^{+/-}$ (**m-o**, respectively) isogenic systems. **p** Number of genes in the major cluster (sub-network) of genes with a decreased correlation in the mutant cells.

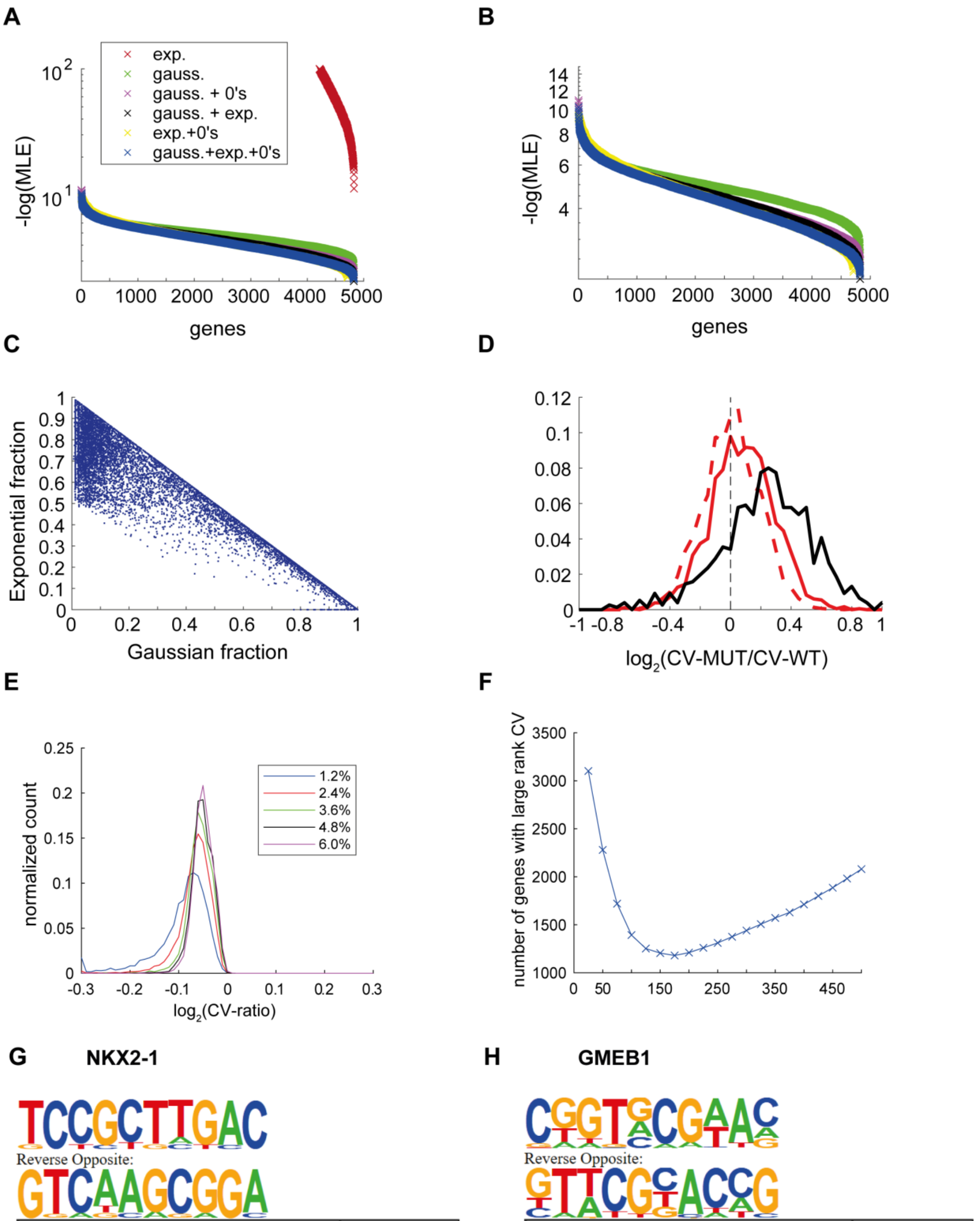

p-value:	1e-10
log p-value:	-2.429e+01
Information Content per bp:	1.844
Number of Target Sequences with motif	13.0
Percentage of Target Sequences with motif	11.30%
Number of Background Sequences with motif	29.0
Percentage of Background Sequences with motif	0.72%

p-value:	1e-10
log p-value:	-2.444e+01
Information Content per bp:	1.700
Number of Target Sequences with motif	18.0
Percentage of Target Sequences with motif	18.18%
Number of Background Sequences with motif	89.0
Percentage of Background Sequences with motif	2.18%

Figure S5. Model fit for gene expression distribution and characterization of differentially-variable genes. Related to Figures 3 and 4. a-b Shown is the (sorted) average log-likelihood of the best fit for 6 different models (see Methods for more details) in the 180Q-corrected cell line: exponential distribution, Gaussian distribution, mixture of Gaussian and uniform zeros distributions, mixture of Gaussian and exponential distributions, mixture of exponential and uniform zeros distributions and mixture of Gaussian, exponential and uniform zeros distributions. The mixture model which uses 3 distributions outperforms the rest of the models. **b** As in (**a**), plotted with better resolution for lower values. **c** The Exponential and Gaussian fractions of the best fir of the 3 distributions mixture model of all expressed genes from the 6 cell lines used in the this study. **d** Same as Figure 3**i** after removal of genes in suspicious chromosomal regions. The distribution of the log ratio of the CV between mutant and WT for the three isogenic systems. p-value for the $CHD^{+/-}$ system in this analysis is << 10^{-60} (Wilcoxon signed rank test). 72Q: dashed red line, 180Q: red solid line, $CHD8^{+/-}$: black solid line. **e** The CV is more stable after trimming the outliers. Shown is the distribution of the log2 ratio of the CV before vs after exclusion of additional 1.2% of the extreme low and high expression values. Extreme 1.2% - blue, 2.4%, 3.6%, 4.8%, 6% in red, green, black and purple, respectively. **f** Rank score stability as a function of the size of the running window. Shown is the number of genes with larger noise (measured by CV) in their calculated rank score (see Methods for more details) above noise thresholds of 0.1. The optimum window size, where the number of genes that succeed the noise threshold is minimal, is achieved around 150 points. **g-h** The enriched known binding motifs in the promoter regions of DV-genes. **g** DV-genes, which are more variable in 180Q mutant cells, are enriched for NKX2-1 binding motif. **h** DV-genes, which are more variable in 180Q-corrected cells, are enriched for GMEB1 binding motif.

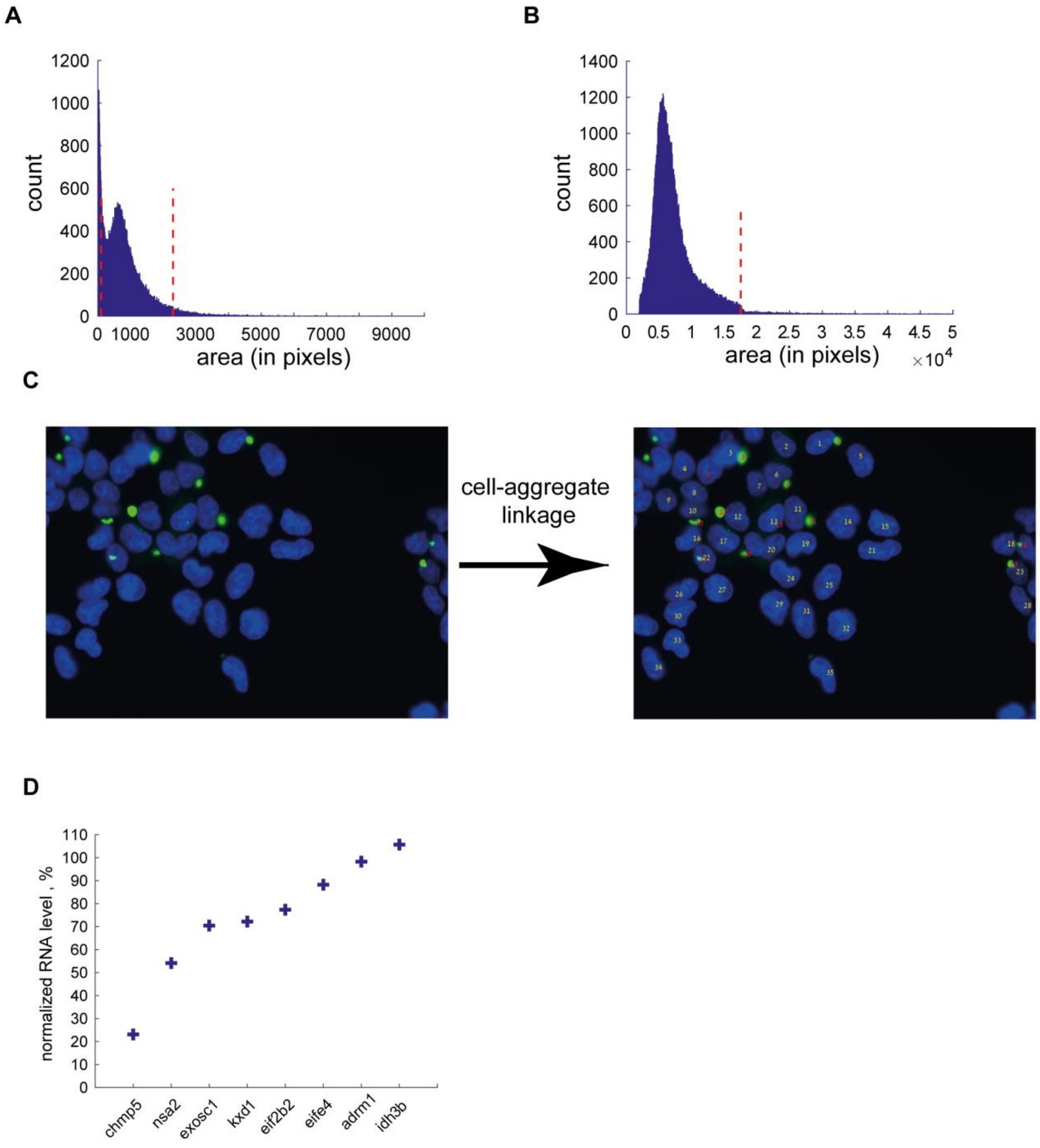

Figure S6. Image analysis of aggregate functional assay. Related to Figure 5. a-b Filtering out suspicious results. **a** Distribution of nuclei area across all images. Nuclei are filtered out if they are too large or too small (thresholds marked by the red dashed line). **b** Distribution of aggregates size across all images. Aggregates are filtered out if they are too large (threshold marked by the red dashed line). **c** At the last stage, identified nuclei and aggregates are linked to each other based on their distance. Numbers on nuclei represent the cell numbering and numbers on aggregates represent the cell number to which the aggregates are assigned. **d** The percentage of RNA levels in different gene knockdown in NPCs. Expression levels are normalized to ACTβ and to the median expression levels in non-treated (WT) and scrambled samples.

Supplementary Methods

Gene correlation comparison:

In order to compare the gene correlations between different conditions, the number of cells must be taken into account. In Figure S4J we used 100 random sampling of X cells and showed that the larger X the narrower the correlation distribution, In Figure S4K we used 50 random sampling of Y cells as initial pools and then randomly sampled 30 cells and calculated the average correlations, to show that the larger Y, the narrower the correlation distribution.

Because the distribution of gene expression correlations is dependent on the number of cells, to compare between WT and mutant cells we used a sub-sampling procedure in which we randomly drew the same number of cells from both conditions and only then calculated the Spearman correlation between every two genes. The number of cells for sub-sampling was chosen as the largest number that divides in 5 and is smaller than the number of cells available in both WT and mutant. We randomly sampled 30 cells out of each of the sub-sampled pools and calculated the Spearman correlations between all genes with average normalized counts larger than 5. This procedure was repeated 1000 times and then averaged, yielding an average correlation between every 2 genes. The procedure of sub-sampling was repeated 10 times to validate that the obtained statistics were not biased as a result of the specific sub-sampled pool that was chosen for the analysis. For the analysis we used only genes that were expressed in at least half of cells in both conditions and had a total average expression of at least 10 normalized counts.

We mark by $C_{i,j}^{wt}$ the spearman correlation between gene i and gene j in WT cells and in $C_{i,j}^{mut}$ the correlation between gene in mutant cells. The average squared correlation of gene i is in condition *cond* is defined as $\overline{(C_i^{cond})^2} = \frac{1}{n}\sum_{j=1}^{n}\left(C_{i,j}^{cond}\right)^2$ where n is the number of genes. The final quenched average squared correlation for gene i is the average of the 10 sub-sampling replicates. The squared correlation difference between two genes i, j is defined as $\left(C_{i,j}^2\right)^{dif} = \left(C_{i,j}^{WT}\right)^2 - \left(C_{i,j}^{mut}\right)^2$. The final quenched squared correlation difference for gene i, j is the average of the 10 sub-sampling replicates.

Mixture model for gene expression data:

To describe the shape of gene expression levels we used a mixture model of three distributions: A Gaussian, an Exponential distribution and a Uniform distribution between 0 and 1. We used Expectation-Maximization (EM) algorithm, which is based on the maximum likelihood criterion, to find the best fit. To avoid local maxima, we used 1000 random initial seeds and chose the best result. The parameters for the fit are: τ_1, τ_2, τ_3 for the relative fraction assigned to the exponential, the normal and the 0's uniform distributions, respectively. λ is the inverse of the mean of the exponential distribution. μ, σ are the mean and standard deviation of the normal distribution, respectively. The EM algorithm repeats the sequence of expectation and maximization steps. Assuming n samples, the expectation step in the (t+1) iteration is defined by:

$$T_1^{i^{(t+1)}} = \frac{\tau_1{}^{(t)} \cdot \lambda^{(t)} e^{-\lambda^{(t)} x_i}}{Z_i{}^{(t)}}, T_2^{i^{(t+1)}} = \frac{\tau_2{}^{(t)} \cdot \frac{1}{\sqrt{2\pi}\sigma^{(t)}} e^{-\left(\frac{\left(x_i - \mu^{(t)}\right)^2}{2\sigma^{(t)^2}}\right)}}{Z_i{}^{(t)}}$$

$$T_3^{i^{(t+1)}} = \frac{\tau_3{}^{(t)} \cdot I_{[0,1]}}{Z_i{}^{(t)}}$$

for i =1,…,n where x_i is the value of the $i'th$ sample, and

$$Z_i{}^{(t)} = \tau_1{}^{(t)} \cdot \lambda^{(t)} e^{-\lambda^{(t)} x_i} + \tau_2{}^{(t)} \cdot \frac{1}{\sqrt{2\pi}\sigma^{(t)}} e^{-\left(\frac{\left(x_i - \mu^{(t)}\right)^2}{2\sigma^{(t)^2}}\right)} + \tau_3{}^{(t)} \cdot I_{[0,1]}$$

with $I_{[0,1]}$ being the indicator function in the range [0,1].

And the M-step is defined by

$$\tau_1{}^{(t+1)} = \frac{1}{n}\sum_{i=1}^{n} T_1^{i^{(t+1)}}, \tau_2{}^{(t+1)} = \frac{1}{n}\sum_{i=1}^{n} T_2^{i^{(t+1)}}, \tau_3{}^{(t+1)} = \frac{1}{n}\sum_{i=1}^{n} T_3^{i^{(t+1)}}$$

$$\lambda^{(t+1)} = \frac{\sum_{i=1}^{n} T_1^{i^{(t+1)}}}{\sum_{i=1}^{n} T_1^{i^{(t+1)}} \cdot x_i}$$

$$\mu^{(t+1)} = \frac{\sum_{i=1}^{n} T_2^{i^{(t+1)}} \cdot x_i}{\sum_{i=1}^{n} T_2^{i^{(t+1)}}}, \sigma^{(t+1)^2} = \frac{\sum_{i=1}^{n} T_2^{i^{(t+1)}} \cdot \left(x_i - \mu^{(t+1)}\right)^2}{\sum_{i=1}^{n} T_2^{i^{(t+1)}}}$$

For technical computational reasons, if during the iterations the best λ is at infinity, we set $\tau_1 = 0$ and add T_1 to T_3.

Image analysis:

We built a separate pipeline in CellProfiler software to identify nuclei and aggregates. For nuclei detection, to better separate between neighboring nuclei, we first used the EnhancedEdges module using the Sobel method to detect nuclei boundaries. The resulting image was further processed using minimum cross entropy with lower bound on threshold=0.1 and upper bound=1 to recognize the borders. These boundaries were then subtracted from the original image. Nuclei were then identified in the corrected image using minimum cross entropy thresholding method with parameters typical diameter=50-150 pixels, scale factor= 1.3488, size of smoothing filter=45, “distinguish clumps by intensity”, and “fill holes after declumping only”. Nuclei that were wrongly divided were then merged using the Merge module with Distance criterion and minimum intensity fraction=0.9, using the “find object intensity with centroids” option. All other parameters in the pipeline were chosen as the default parameters.

For aggregate detection in HEK cells we used adaptive thresholding with the Otsu method with three classes-thresholding with parameters: typical diameter=5-100 pixels, threshold smoothing scale=3.5, lower and upper bounds on threshold=0.2-1, size of adaptive window=100, method to distinguish clumped objects=shape, using “fill holes after both thresholding and declumping”. This was followed by Merge module to merge detected objects using the Distance criterion. Next, to remove artifacts, detected objects were filtered out if they were not round using the criterion of a form factor larger than 0.75. All other parameters in the pipeline were chosen as the default parameters. Following image analysis, nuclei were filtered out if they were too large (area > 17500 pixels) and aggregates were filtered out if they were too large (area > 2300 pixels) or too small (area < 100 pixels) (Figure S5A-B). Aggregates were then linked to the closest nucleus in the image. Based on the empirical distribution of the distances between aggregates and their closest nuclei (Figure S5C), aggregates were removed from the analysis if this distance was larger than twice the mode of the distribution (threshold = distance of 104 pixels).

For NPC aggregate detection, we used Global Manual thresholding using “manual threshold=0.4” and typical diameter=5-100 pixels, and method to distinguish clumped objects=shape, using “fill holes after both thresholding and declumping”. This was followed by Merge module to merge detected objects using the Distance criterion. Following image analysis, nuclei were filtered out if they were too large (area > 10,000 pixels) and aggregates were filtered out if they were too large (area > 2500 pixels). Aggregates were then linked to the closest nucleus in the image. Based on the empirical distribution of the distances between aggregates and their closest nuclei, aggregates were removed from the analysis if this distance was larger than 70 pixels.
