# Supplementary material for: Pluripotent stem cell-derived models of neurological diseases reveal early transcriptional heterogeneity
Source: Genome Biol. 2021 Mar 4;22:73. doi: 10.1186/s13059-021-02301-6 (PMC7934477; doi:10.1186/s13059-021-02301-6)
Supplement: Supplementary file 6 — Additional file 6. Review history. [file 13059_2021_2301_MOESM6_ESM.docx]

Review History

**First round of review**

**Reviewer 1**

**Were you able to assess all statistics in the manuscript, including the appropriateness of statistical tests used?**

No, I do not feel adequately qualified to assess the statistics.

**Comments to author:**

In the manuscript: "Pluripotent stem cell derived models of neurological diseases reveal early transcriptional heterogeneity" Sorek et. al report that transcriptional heterogeneity which presumably precedes neurodegeneration might be a feature of several distinct neurodegenerative (AD, HD) or even neuropsychiatric diseases (ASD). They conclude that the reported transcriptional heterogeneity might be useful as a diagnostic tool or for future interventions. This is a topic of high significance and the paper should be of broad interest to others in the field of neurodegeneration and aging. Methods that could lead to early diagnosis or early therapies are desperately needed.

The authors have broad expertise in the analysis of scRNA seq data and employ state-of-the art scRNA-Seq using a modified Smart-seq2 protocol in the new experiments. Overall, the methods employed are well described. However, we think that several aspects of the manuscript require additional experiments and clarifications to test/support the hypothesis that transcriptional heterogeneity as a common theme across many diseases is causal and not consequential to other phenotypes or cell death.

1. The AD single-cell seq study. Insufficient data was provided on how the cellular diversity was handled. I presumed, based on previous publications, that cell-type pathology was driven by microglia, oligodendrocytes, and astrocytes. Can the authors analyze transcriptional heterogeneity in these cells? This should be important.

2. The authors say that they validated that the contribution of the different neuron sub-types was similar among AD and healthy subjects. We expected differences based on broad degeneration in AD brains and the believe that death of neurons is not purely stochastic but shows preference, especially early vulnerability in distinct regions. We were not able to understand how the authors determined similarity. This is important as a survival bias could greatly affect the observed heterogeneity. For example, if a specific population of excitatory neurons that makes up a large proportion of the excitatory neurons dies preferentially the remaining populations of excitatory neurons could appear more diverse. The authors should present scRNA seq data focusing and relevant plots looking only at excitatory neurons using established expression clusters to guide this analysis.

3. Choice of models. It is really unclear to me how the authors go from the AD study to the HD study. Was this driven by the availability of the model? The diseases and known mechanisms are so different. HD onset can be early, even in children, and typically manifest around age 40. AD onset is late. Known pathological mechanism are distinct. I do not know much about the ASD model, but again there is not much of a connection. I believe that genes that cause neurodegeneration or neuropsychiatric disease that affect specific neurons or brain regions could have other, milder phenotypes, with accompanying changes in transcriptional patterns, primary dysregulation or secondary to other phenotypes in other unrelated cell types. It is unclear how relevant this would be to disease.

4. Transcriptional heterogeneity as a tool for early diagnosis. Is the observable heterogeneity predictive? If yes, this should be explained better.

5. Problems with the in vitro model and differentiation model. Little characterization of the differentiated cell types was offered. In the provided supplemental figure S2. The human pluripotent stem cells in S2A d0 do not show the expected colony formation with the typical morphology of cells (round cells, large nucleus, tiny ring of cytoplasm) The colonies do not show smooth edges with well-defined borders). Maybe the cells were kept in Rock-inhibitor conditions, the morphology looks similar, but the authors should explain. It would be good to see all four cell lines in comparison. Is this morphology a phenotype in pluripotent stem cells?

It is unclear how the experiments were performed. This is especially important to judge the expected variation. Were these three independent differentiation experiments, conducted at distinct times? Or technical replicates?

The staining data provided in the manuscript in supplemental figure 2SB raised concerns: TUJ1 is a marker for neurons. It is often used to distinguish neural progenitor cells from neurons. For the NPC stain I would recommend Nestin and contrast this with a TUJ1 stain. I would expect both NPCs and early neurons in the cultures at day 20. The picture of the stained "NPCs" does not look similar to the provided brightfield picture. Overall, this characterization is not acceptable.

If the experiments are repeated, I would recommend showing data from all cell lines during differentiation and quantification of any changes in percentage of TUJ1, NESTIN positive cells, any observable phenotypes etc to judge the impact on observed heterogeneity.

If experiments are repeated, I recommend utilizing a high-efficiency method to generate excitatory neurons using NGN2 overexpression in all cell lines. This will remove a lot of the differentiation bias and will allow the authors to focus on the observable transcriptional heterogeneity. This could be contrasted with a high efficiency programming method to generate GABAergic neurons. This experiment will be very informative, greatly reduce differentiation variability and is not difficult to perform.

6. Validation experiments in HEK cells. It is unclear why the validation experiments were carried out in HEK cells? It is unclear how the specific genes were picked from the pool of available DV genes. I would expect aggregation of the GFP-105Q if lysosomal or proteasomal functions are negatively perturbed. Level of knockdown is unclear and how this compares to the effect size seen in DVs? Couldn't these experiments be performed in differentiated cells? Ideally again in NGN2 programmed excitatory neurons. These experiments might be more meaningful as comparisons between 180Q and isogenic revertant both expressing the GFP-105Q. Perturb genes in revertants to see if they reach the level of aggregation seen in 180Q cell lines which should be the baseline.

7. I am looking forward to the revised version of the manuscript and I hope that the authors can perform additional experiments to strengthen this important study.

**Reviewer 2**

**Were you able to assess all statistics in the manuscript, including the appropriateness of statistical tests used?**

Yes, and I have assessed the statistics in my report.

**Comments to author:**

Sorek and colleagues use post-mortem Alzheimer's disease (AD) single brain cell RNAseq data, together with three neurodegenerative disease (ND) models: HTT 72 polyQ, HTT 180 polyQ (HD) and CHD8 (ASD), to investigate transcriptional variation across cells. Using mRNA measurements they attempt to investigate two hypotheses. The first, is that neuronal death in NDs due to genetic defects that directly perturb cellular function, by for example increasing protein aggregate formation (HD). Alternatively, neuronal death is a probabilistic (stochastic) effect of a genetic detect, which is compensated by the neuron at the expense of other homeostatic regulation. The additional / diverted resources expended on homeostasis make ND neurons more susceptible to death, especially with aging.

The authors use transcriptomic variance estimation, transcriptomic network analysis, and experimental knock-down of hypervariable genes in cell culture to advance the hypothesis that neuronal death is stochastic rather than deterministic in several NDs.

Unfortunately there are several major limitations with the current manuscript in both its broader hypotheses and statistical approaches.

The idea that transcriptional repertoires in iPSC derived ND neurons will show greater transcriptional variation as well as differential expression (differences in mean) is an interesting hypothesis which most researchers would probably accept as likely true, but shy away from testing due to the difficulty in investigating this. It is not clear why the team has chosen these particular ND models for this investigation.

It is unclear that transcriptional variation can be proffered as evidence to prefer one model of neurodegenerative disease aetiology over another. The toxic molecules in HD and AD are proteins, not RNA. Further, unlike in AD and HD, there is no gross morphological cell ‘damage’ in ASD, merely abnormal neuronal morphology in certain ASD forms. To test the competing hypotheses, a phenotype more reflective of the disease state, for example the number of Huntingtin inclusions per HD cell / amyloid inclusions per AD cell, or another measure of cellular viability, would have to be correlated with the transcriptomic state of each cell. Even then, work would be required to test whether the unhealthy cells had arisen due to direct, or stochastic effects of the relevant genetic lesion.

Part of the difficulty with framing the work around the deterministic/stochastic dichotomy, is that the background context of perturbed C. elegans neurite migration in a triple-knockout animal, is a developmental phenotype unrelated to cellular ‘damage’ or aging, and thus mostly irrelevant for the NDs in question.

Lastly, as mentioned above dysregulated transcription is expected in NDs due to the generally degraded neuronal health. Given the germline origin of HD mutations in particular, it is difficult to distinguish correlation from causation in this manuscript, and to see the utility of neuronal transcriptomic variance as a putative disease marker.

The HD model is particularly inappropriate for investigation of variation given that there is greater expression variability expected in neuronal cells due to the somatic mutation rate/instability of the repeat. This will be higher than for a point mutation or simple K/O and is even more atypical to more general neurological models.

A time-course analysis combining imaging or other single-cell phenotyping with sc transcriptomics might be the best approach for addressing this research question.

The authors use scRNAseq and bulk RNAseq data to investigate this hypothesis using a variety of statistical approaches to try and estimate variation. Estimating variation and testing for differential variation is difficult as it has to be disentangled from known changes in means (standard differential expression analysis).

The authors show some interesting results but overall the paper is not convincing in its premise. This is also due to the limitations of the three models used and the difficulties with the statistical approaches used. The authors need to perform more analyses, especially under the null hypothesis, to convince readers that their statistical analysis approaches are sound and robust. To this end, a series of analysis with their methods under a variety of known null results should be performed. This should include: (i) permuted label analysis of WT and mutations in all three models, and (ii) WT-only analyses to ensure that unexpected results do not occur.

The authors attempt to validate some of their results using a reporter assay but the results are not quantitatively described and two genes only are shown as controls, without any validation.

More detail is required for the cell lines and their driver mutations for HTT and CHD8.

In terms of statistics, the p values quoted for multiple t test results are below 10E-100, which is simply not plausible given the overlap in distributions e.g. in Figure 2B. More detail in methods, and a revision of the test results is required.

Minor comments

The figure quality & resolution in the pdf is very poor. This is the case for all five figures.

P-values are reported without naming tests, or inadequate description of test used, and whether assumptions have been met.

Ln 278-280 ‘This model also has computational

advantages, as the NB model has no closed formula for the maximum-likelihood solution,

which is even more difficult when a mixture-model parameters are to be deduced.’

That’s not a problem with the many computational approaches to estimating parameters via ML by using hybrid approaches with the EM, although, of course, less elegant to the EM presented by the authors in the supplementary methods..

Ln 285 “could be dominantly fitted by”. What do you mean by dominantly fitted? Best fit? How is this determined?

Ln 341 “to reversely correlate” “to inversely correlate”?

Ln 379 No such motif was found in the CHD8 model?

Ln 410 why/how were the two control genes chosen? It would be good to see results for a few more control genes.

Ln 411 how many constitute the “great majority” ? This is very imprecise. What statistical threshold was used?

L436 Instead of using the phrase “investigating the time dimension” it would be more appropriate to say “investigate the age of onset and hence severity”

L503 “titer”

L509 “imbalance with three…”

L570 “ snRNAseq” should be “scRNAseq”?

L580 Please state whether paired end and read length, as well as targeted lib size.

L600 “Cells that had an N-E index smaller than 2 were discarded from further analysis.” Please demonstrate that this index was reasonable and robust to discriminate cells. Fig S3-F displays this ratio but it still seems a very arbitrary cut off with no clear separation.

L641 Specify the total number of parameters for this model: 2 + 2 + 1 ? 2 from the weights/proportions of the three models, two for the normal/gaussian and one for the exponential.

L648 “ For the DVSM analysis, only genes with average expression larger than 5 normalized counts “ – It is not clear by what you mean here. Also, don’t you mean variation?

L655 Why is this a 3 parameter model? Are you assuming mu = 0, sigma = 1???

How good is the fit of these models? Please demonstrate through simulations and on your results – QQ plots etc. If the fitting is nonsense then the following analysis which makes use of the gaussian weights will be non-sense too.

F1 legend – p-value without test statistic.

L763 define “GRN”

L769 explain: “Error bars represent 1 std over 10 replicates (see Figure S4 and Methods).”

FigS3G – There seem to be other cells that are also showing signals of being outliers very similar to the outlier that was removed. (at 15 & 55).

**Authors Response**

**Point-by-point responses to the reviewers’ comments:**

Reviewer #1:

In the manuscript: "Pluripotent stem cell derived models of neurological diseases reveal early transcriptional heterogeneity" Sorek et. al report that transcriptional heterogeneity which presumably precedes neurodegeneration might be a feature of several distinct neurodegenerative (AD, HD) or even neuropsychiatric diseases (ASD). They conclude that the reported transcriptional heterogeneity might be useful as a diagnostic tool or for future interventions. This is a topic of high significance and the paper should be of broad interest to others in the field of neurodegeneration and aging. Methods that could lead to early diagnosis or early therapies are desperately needed.

- Thank you very much.

The authors have broad expertise in the analysis of scRNA seq data and employ state-of-the art scRNA-Seq using a modified Smart-seq2 protocol in the new experiments. Overall, the methods employed are well described. However, we think that several aspects of the manuscript require additional experiments and clarifications to test/support the hypothesis that transcriptional heterogeneity as a common theme across many diseases is causal and not consequential to other phenotypes or cell death.

- We appreciate the Reviewer’s careful reading and constructive comments. We provide a detailed point-by-point response addressing the Reviewer’s concerns.

1. The AD single-cell seq study. Insufficient data was provided on how the cellular diversity was handled. I presumed, based on previous publications, that cell-type pathology was driven by microglia, oligodendrocytes, and astrocytes. Can the authors analyze transcriptional heterogeneity in these cells? This should be important.

*- We thank the Reviewer for this comment. Incidentally, it allowed us to test our hypothesis in two separate datasets, one in which cellular composition was unaltered, and one in which cellular composition was significantly different. Comparing the cell composition in the Mathys et al. data, we observed no difference in the cellular composition between healthy and AD patients (Figure R1 A). In contrast, in the Grubman et al. data, there is a significant difference in cell composition, with significantly more oligodendrocytes in AD patients compared to healthy controls, at the expense of neurons, astrocytes and OPCs (Figure R1 B). While we do appreciate that this is important by itself for understanding disease mechanisms, our analysis examined separately each of these cell types between AD and healthy patients, ignoring the differences in numbers. Hence, we now provide evidence supporting increased heterogeneity in AD regardless of whether cellular composition is altered and regardless of the dataset used.*

*Figure R1:*

*A) The cellular composition of the main brain cell types (from Mathys et al., 2009) among 24 healthy and 24 AD patients is similar. Two-tailed t-test for excitatory (Ex), inhibitory (In), astrocytes (Ast), microglia (Mic) and oligodendrocytes (Oilgo) were all non-significant (p= 0.869, 0.473, 0.576, 0.492, and 0.877, respectively).*

*B) Same analysis for data from Grubman et al., 2019. P-values for neurons, microglia, oligodendrocytes, endothelial cells (Endo), astrocytes and oligodendrocyte progenitor cells (Opc) are 0.09, 0.45, 0.0014, 0.94, 0.0044 and 4.59E-4, respectively.*

*The reason we did not include data from the non-neuronal cell types in the original submission was due to their small numbers. As can be seen below (Figure R2 A-B: median percentage of cells expressing the 200 most highly expressed genes), both AD (red) and controls (blue) had too few cells to reach significance.*

*Same is true for the Grubman et al. data: the median number of microglia cells was only 34 and 25 in healthy subjects and AD patients, respectively, and 7 and 2.5 for endothelial cells. For the rest of the cell types, there is an effect, albeit not significant, due to the small number of patients (Figure R2 C-E).*

*Following the reviewer’s comment, we now added these results to Figure S1 as panels h-j.*

*Figure R2:*

*A-B) The median percentage of cells expressing the 200 most highly expressed genes in Microglia (A), p = 0.8708 and Oligodendrocytes (B), p = 0.2611 from Mathys et al.*

*C-E) The median percentage of cells expressing the 100 most highly expressed genes in OPC (C), p = 0.2, Astrocytes (D), p = 0.2 and Oligodendrocytes (E), p = 0.87 from Grubman et al. All p-values are based on Wilcoxon rank-sum test.*

2. The authors say that they validated that the contribution of the different neuron sub-types was similar among AD and healthy subjects. We expected differences based on broad degeneration in AD brains and the believe that death of neurons is not purely stochastic but shows preference, especially early vulnerability in distinct regions. We were not able to understand how the authors determined similarity. This is important as a survival bias could greatly affect the observed heterogeneity. For example, if a specific population of excitatory neurons that makes up a large proportion of the excitatory neurons dies preferentially the remaining populations of excitatory neurons could appear more diverse. The authors should present scRNA seq data focusing and relevant plots looking only at excitatory neurons using established expression clusters to guide this analysis.

*- As shown in Figure R1 B, the data in Grubman et al. supports the notion that there is an AD-related shift in the relative composition of cells in the brain. In contrast, the larger, Mathys et al. data, does not show any difference in the contribution of the main cell types (Figure R1 A). In these data the authors have also used the large number of cells to annotate the different cellular sub-types. This includes 13 subtypes of excitatory neurons, 12 subtypes of inhibitory neurons, 5 subtypes of oligodendrocytes, and 4 subtypes of microglia and astrocytes. Using this annotation, we compared the relative contribution of these subtypes to AD and control subjects. We found no difference in the cellular composition of each main neuronal type for all subtypes (Table R1). We also applied this analysis to the joined group of excitatory and inhibitory neurons (total 25 sub-types) and found no difference. Therefore, the analysis can be done at the level of the main neuronal types, thus exploiting the large number of cells to be able to compare between healthy and AD subjects.*

*Table R1*

3. Choice of models. It is really unclear to me how the authors go from the AD study to the HD study. Was this driven by the availability of the model? The diseases and known mechanisms are so different. HD onset can be early, even in children, and typically manifest around age 40. AD onset is late. Known pathological mechanism are distinct. I do not know much about the ASD model, but again there is not much of a connection. I believe that genes that cause neurodegeneration or neuropsychiatric disease that affect specific neurons or brain regions could have other, milder phenotypes, with accompanying changes in transcriptional patterns, primary dysregulation or secondary to other phenotypes in other unrelated cell types. It is unclear how relevant this would be to disease.

*- The hypothesis we present is general for NDs. We used the AD single cell data because it was available and it allowed us to readily test our hypothesis using existing data. The advantage of AD as a model is that the cells analyzed were naturally obtained years after the disease onset, and therefore the phenotype of transcriptional variability is more likely to be observed compared to ‘younger’ cells. However, the AD model has two main shortcomings: First, our hypothesis is that transcriptional variability is an earlier phenotype that precedes disease onset which cannot be tested in the AD cells, and cannot be done prior to disease onset (in vivo) for as long as we do not have a definite genetic mutation that causes AD. Second, the AD cells come from different genetic backgrounds (i.e. different subjects) and therefore it is harder to separate between genetic and non-genetic transcriptional variability.*

*Therefore, to test our hypothesis more directly, we used the HD and ASD models, where, in both cases, we have a causal mutation that predicts the disease with full penetrance, and with similar genetic backgrounds (as we use isogenic systems). In addition, we could manipulate the iPSC cells in culture to NPCs, a relatively homogeneous cell type. The main original caveat of our iPSC-derived NPCs is that our cells are ‘young’, equivalent to an early neuronal state, which might have been too early to observe changes in transcriptional heterogeneity. Following the reviewer’s comment, we added text in the manuscript in the Background section (P. 6) to better clarify this logic.*

4. Transcriptional heterogeneity as a tool for early diagnosis. Is the observable heterogeneity predictive? If yes, this should be explained better.

*- Our manuscript indeed suggests that the answer to this question is yes. We added a paragraph that explains this point in the Discussion (P. 25).*

5. Problems with the in vitro model and differentiation model. Little characterization of the differentiated cell types was offered. In the provided supplemental figure S2. The human pluripotent stem cells in S2A d0 do not show the expected colony formation with the typical morphology of cells (round cells, large nucleus, tiny ring of cytoplasm) The colonies do not show smooth edges with well-defined borders). Maybe the cells were kept in Rock-inhibitor conditions, the morphology looks similar, but the authors should explain. It would be good to see all four cell lines in comparison. Is this morphology a phenotype in pluripotent stem cells?

*- We refer to the beginning of the differentiation as d0. We apologize for not making it clear. The presented cells marked as d0 are cells from d3, grown on matrigel and after Rock inhibitor treatment for the first 2 days. Following the reviewer’s comment, we added images along the differentiation process for all six cell lines (four HD-related lines and two ASD-related lines), and updated Figure S2:*

*Figure R3 (and revised Figure S2). Experimental setup of scRNA-Seq of neuronal progenitor cells in culture. Related to Methods.*

*A) During differentiation, cells gradually acquire neuronal identity. Shown are days of selected stages of the protocol. Scale bars = 50 μm*

*B) Overview of the neuronal differentiation protocol of pluripotent stem cells*

*C) NPCs express the neuronal marker TUJ1. Shown are example of the mutant and corrected 72Q cells. Scale bars = 10 μm*

*D) Our experimental pipeline.*

It is unclear how the experiments were performed. This is especially important to judge the expected variation. Were these three independent differentiation experiments, conducted at distinct times? Or technical replicates?

*- These were independent experiments. Following the reviewer’s comment, we added this information in the Methods section (P. 27).*

The staining data provided in the manuscript in supplemental figure 2SB raised concerns: TUJ1 is a marker for neurons. It is often used to distinguish neural progenitor cells from neurons. For the NPC stain I would recommend Nestin and contrast this with a TUJ1 stain. I would expect both NPCs and early neurons in the cultures at day 20. The picture of the stained "NPCs" does not look similar to the provided brightfield picture. Overall, this characterization is not acceptable.

If the experiments are repeated, I would recommend showing data from all cell lines during differentiation and quantification of any changes in percentage of TUJ1, NESTIN positive cells, any observable phenotypes etc to judge the impact on observed heterogeneity.

*- We used cells which have already gone through neuronal commitment but are not yet fully differentiated to mature neurons (Figure R4). In order to achieve this state, we validated that the neuronal commitment marker TUJ1 is expressed in the cells. In addition, we also sorted the cells using PSA-NCAM, an early marker of neural differentiation. The overlap between PSA-NCAM and TUJ1 defines a specific differentiation window of “immature neurons” (Figure R4). This filters out cells that have already further advanced along the neuronal differentiation axis, but at the same time ensures neuronal commitment. The cells presented in the bright field images are taken at 20X magnification where the stained NPC is taken at 60X and cells were grown on glass, where they are less dense.*

*Figure R4. Expression of different markers during neuronal differentiation. Image taken from “Adult hippocampal neurogenesis is abundant in neurologically healthy subjects and drops sharply in patients with Alzheimer’s disease.” Moreno-Jiménez EP et al. Nature Medicine, 2019.*

If experiments are repeated, I recommend utilizing a high-efficiency method to generate excitatory neurons using NGN2 overexpression in all cell lines. This will remove a lot of the differentiation bias and will allow the authors to focus on the observable transcriptional heterogeneity. This could be contrasted with a high efficiency programming method to generate GABAergic neurons. This experiment will be very informative, greatly reduce differentiation variability and is not difficult to perform.

*- As explained above, we were able to achieve a fairly homogenous cell population (of “immature neurons”) using the two-marker approach described (Figure R4). We also differentiated the iPSCs towards post-mitotic striatal GABAergic medium spiny neurons (MSNs), but the SMaRT-Seq2 single-cell RNA-seq experiments proved too challenging for these cells. In general, the more differentiated the neurons are, the more sensitive they become. Staining our iPSC’s derived MSNs using the apoptosis marker Annexin-V and PI for dead cells we observed that most (>95%) of these cells become heavily damaged shortly after the cells are removed from culture, which introduces a serious technical limitation.*

6. Validation experiments in HEK cells. It is unclear why the validation experiments were carried out in HEK cells?

*- We have carried out the initial validation in HEK cells because they are human cells for which we had a simple and elegant system for the readout of cellular pathology (i.e., aggregates marked with GFP) and for which we could easily perform large-scale imaging experiments. However, following the reviewer’s comment, in recent months we have developed a similar system in 180Q NPCs (from Huntington’s disease 180Q iPSCs) that also form aggregates. We repeated the screen using more genes in three biological replicates. We calibrated our HEK screen image analysis pipeline to the new NPC images and repeated the analysis of aggregate quantification in the different gene knockdowns (Figure R5). Reassuringly, we found, once again, that most knockdowns of DV genes resulted in increased aggregate formation. We added the new results to the revised manuscript (Figure 5G-H in the manuscript, and main text: PP. 7; 19-20; 23).*

*Figure R5. Functional validation assay for HD potentially-related genes in NPCs.*

*A) Percentage of NPC cells, which have at least one polyQ aggregate, following knockdown of different target genes (indicated below). Red boxes represent DV genes with increased variability in mutant cells and black boxes represent control genes. Green dashed line represents the average of 2 standard errors of the mean from the average WT (non-treated) and SC (scrambled) samples.*

*B) Total number of cells (stained nuclei) for each target gene in the siRNA experiment.*

A) Percentage of NPC cells, which have at least one polyQ aggregate, following knockdown of different target genes (indicated below). Red boxes represent DV genes with increased variability in mutant cells and black boxes represent control genes. Green dashed line represents the average of 2 standard errors of the mean from the average WT (non-treated) and SC (scrambled) samples.

B) Total number of cells (stained nuclei) for each target gene in the siRNA experiment.

It is unclear how the specific genes were picked from the pool of available DV genes.

*- We chose the DVSM genes that had the largest effect (CV ratio) in both the 180Q and the 72Q systems, or at least in the 180Q system. Following the reviewer’s comment, we added a text in the manuscript clarifying this point (P. 18).*

*Figure R6 (Figure S6 in the revised manuscript): The percentage of RNA levels in gene knockdown compared to non-treated (WT) and scrambled knockdown.*

I would expect aggregation of the GFP-105Q if lysosomal or proteasomal functions are negatively perturbed. Level of knockdown is unclear and how this compares to the effect size seen in DVs? Couldn't these experiments be performed in differentiated cells? Ideally again in NGN2 programmed excitatory neurons. These experiments might be more meaningful as comparisons between 180Q and isogenic revertant both expressing the GFP-105Q. Perturb genes in revertants to see if they reach the level of aggregation seen in 180Q cell lines which should be the baseline.

*- As explained in the previous point, we repeated the experiment and the downstream analysis in a system of differentiated cells, i.e. NPCs. Following the reviewer’s comment, we also validated the knockdown effect in 8 selected genes. In 6/8 genes tested in real-time qPCR we found a decrease of between 15% to 80% compared to WT and scrambled controls (Figure R6). We also added this result as Figure S6D.*

7. I am looking forward to the revised version of the manuscript and I hope that the authors can perform additional experiments to strengthen this important study.

*- Thank you! We hope and trust the reviewer will find our revised version worthy of publication.*

Reviewer #2:

Sorek and colleagues use post-mortem Alzheimer's disease (AD) single brain cell RNAseq data, together with three neurodegenerative disease (ND) models: HTT 72 polyQ, HTT 180 polyQ (HD) and CHD8 (ASD), to investigate transcriptional variation across cells. Using mRNA measurements they attempt to investigate two hypotheses. The first, is that neuronal death in NDs due to genetic defects that directly perturb cellular function, by for example increasing protein aggregate formation (HD). Alternatively, neuronal death is a probabilistic (stochastic) effect of a genetic detect, which is compensated by the neuron at the expense of other homeostatic regulation. The additional / diverted resources expended on homeostasis make ND neurons more susceptible to death, especially with aging.

The authors use transcriptomic variance estimation, transcriptomic network analysis, and experimental knock-down of hypervariable genes in cell culture to advance the hypothesis that neuronal death is stochastic rather than deterministic in several NDs.

Unfortunately there are several major limitations with the current manuscript in both its broader hypotheses and statistical approaches.

The idea that transcriptional repertoires in iPSC derived ND neurons will show greater transcriptional variation as well as differential expression (differences in mean) is an interesting hypothesis which most researchers would probably accept as likely true, but shy away from testing due to the difficulty in investigating this. It is not clear why the team has chosen these particular ND models for this investigation.

*- As we described in our answer to reviewer 1 above, the hypothesis we present is general for NDs. We used the AD single cell data because it was available and it allowed us to readily test our hypothesis using existing data. The advantage of AD as a model is that the cells analyzed were naturally obtained years after the disease onset, and therefore the phenotype of transcriptional variability is more likely to be observed compared to ‘younger’ cells. However, the AD model has two main shortcomings: First, our hypothesis is that transcriptional variability is an earlier phenotype that precedes disease onset which cannot be tested in the AD cells, and cannot be done prior to disease onset (in vivo) for as long as we do not have a definite genetic mutation that causes AD. Second, the AD cells come from different genetic backgrounds (i.e. different subjects) and therefore it is harder to separate between genetic and non-genetic transcriptional variability.*

*Therefore, to test our hypothesis more directly, we used the HD and ASD models, where, in both cases, we have a causal mutation that predicts the disease with full penetrance, and with similar genetic backgrounds (as we use isogenic systems). In addition, we could manipulate the iPSC cells in culture to NPCs, a relatively homogeneous cell type. The main original caveat of our iPSC-derived NPCs is that our cells are ‘young’, equivalent to an early neuronal state, which might have been too early to observe changes in transcriptional heterogeneity. Following the reviewer’s comment, we added text in the manuscript in the Background section (P. 6) to better clarify this logic.*

*It is unclear that transcriptional variation can be proffered as evidence to prefer one model of neurodegenerative disease aetiology over another. The toxic molecules in HD and AD are proteins, not RNA.*

Further, unlike in AD and HD, there is no gross morphological cell ‘damage’ in ASD, merely abnormal neuronal morphology in certain ASD forms. To test the competing hypotheses, a phenotype more reflective of the disease state, for example the number of Huntingtin inclusions per HD cell / amyloid inclusions per AD cell, or another measure of cellular viability, would have to be correlated with the transcriptomic state of each cell. Even then, work would be required to test whether the unhealthy cells had arisen due to direct, or stochastic effects of the relevant genetic lesion.

*- We did not claim that the transcriptional variability is a better cellular marker than other known disease aetiologies. Our results suggest, however, that transcriptional variability precedes other known cellular phenotypes such as cellular aggregates in HD, and hence it is very valuable. Accordingly, we also added a paragraph discussing the diagnostic potential of transcriptional variability in NDs in the Discussion (P. 25).*

*In the AD model, the cells are in a late state, after disease-onset. In contrast, both in our HD and CHD8+/- models, the relevant proteins are thought to (among other things) regulate transcription, and therefore the increase in transcriptional heterogeneity is a plausible mechanism. It is always possible that there is some cellular “variable” that changes in a deterministic fashion as a result of the mutation, but we show that at least at the level of transcriptional changes, the cells show a pattern that corresponds to stochastic changes.*

Lastly, as mentioned above dysregulated transcription is expected in NDs due to the generally degraded neuronal health. Given the germline origin of HD mutations in particular, it is difficult to distinguish correlation from causation in this manuscript, and to see the utility of neuronal transcriptomic variance as a putative disease marker.

*- We agree with the reviewer that we did not show that transcriptional variability causes the ND phenotype. We suggest that transcriptional heterogeneity precedes the cellular and behavioural phenotypes in HD, where the mutations (in both HD and ASD) are the causal variable, which is responsible for cellular variability as well as other phenotypes. Our results do support, however, a model where the transcriptional variability phenotype is related to the disease phenotypes, where in both the HEK and (the newly added) NPC cellular aggregate reporter system, an intervention in the expression levels of genes with increased transcriptional variability in the disease state results in an increased disease-related phenotypes.*

The HD model is particularly inappropriate for investigation of variation given that there is greater expression variability expected in neuronal cells due to the somatic mutation rate/instability of the repeat. This will be higher than for a point mutation or simple K/O and is even more atypical to more general neurological models.

*- As the reviewer suggested, it is possible that some of the observed variability is related to somatic mutations. Estimating this heterogeneity is extremely hard as repeats are difficult to estimate using PCR. We did not grow the cells for many passages, limiting the potential contribution of somatic mutations, however we cannot exclude the possibility of such an effect. Using HD as a model, however, has other advantages, as it is a late onset neurodegenerative disease, which is caused by one known gene, and we used two independent isogenic systems with different repeat lengths. In addition, our ASD model has a heterozygous KO mutation, which is not inclined for relevant somatic mutations, and still shows the same effect of increased variability, attesting that the somatic mutations in HD are not the whole story.*

A time-course analysis combining imaging or other single-cell phenotyping with sc transcriptomics might be the best approach for addressing this research question.

*- Unfortunately, this is currently technically impossible.*

The authors use scRNAseq and bulk RNAseq data to investigate this hypothesis using a variety of statistical approaches to try and estimate variation. Estimating variation and testing for differential variation is difficult as it has to be disentangled from known changes in means (standard differential expression analysis).

*- We completely agree with the reviewer; it is difficult to disentangle variation from known changes in the mean. To overcome these difficulties, we used two approaches: first, we focused on the genes that have a similar average expression levels in both mutated and control cells. In these genes there is no bias in the variability that corresponds to the mean differences, and therefore a direct comparison between their variability measure is valid. We used both the CV measure and the model-based measure for variability and obtained similar results. Second, to compare variation among all genes including those that have a different average expression in the mutant vs control cells, we first fitted a line to the CV-mean bias and took the residual effect each gene has beyond this expected inherent bias (Figure 4A in the manuscript). This way, we are left with comparable variability measures, which correspond to the variability component which is independent of the mean (Figure 4B in the manuscript) .*

The authors show some interesting results but overall the paper is not convincing in its premise. This is also due to the limitations of the three models used and the difficulties with the statistical approaches used. The authors need to perform more analyses, especially under the null hypothesis, to convince readers that their statistical analysis approaches are sound and robust. To this end, a series of analysis with their methods under a variety of known null results should be performed. This should include: (i) permuted label analysis of WT and mutations in all three models, and (ii) WT-only analyses to ensure that unexpected results do not occur.

*- Following the reviewer’s comment, we added a Supplementary Table (S4), which contains the genes that have a similar average expression levels in the three isogenic systems, along with their corresponding trimmed-CV values in WT and mutant cells and p-values using two different approaches:*

*1. Using a bootstrap approach: expression values are re-sampled from the available expression values for WT and mutant cells separately, generating a confidence interval for the ratio of the trimmed-CV values (mutant divided by WT), which is quantified by the 5th and 95th percentiles of the 10,000 repetitions of the bootstrap procedure. Then, the p-value for ratio>1 (<1) is the % of bootstrap samples where the ratio is <1 (>1).*

*2. Using a permutation test, where the labels of the cells are permuted and the CV ratio is calculated again. Using 10,000 random permutations, the p-value of CV-ratio>1 (<1) is the % of permutations where the CV-ratio is <1 (>1).*

The authors attempt to validate some of their results using a reporter assay but the results are not quantitatively described and two genes only are shown as controls, without any validation.

*- We have carried out the initial validation in HEK cells because they are human cells for which we had a simple and elegant system for the readout of cellular pathology (i.e., aggregates marked with GFP) and for which we could easily perform large-scale imaging experiments. However, following the reviewer’s comment, in recent months we have developed a similar system in 180Q NPCs (from Huntington’s disease 180Q iPSCs) that also form aggregates. We repeated the screen using more genes in three biological replicates. We calibrated our HEK screen image analysis pipeline to the new NPC images and repeated the analysis of aggregate quantification in the different gene knockdowns (Figure R5). Reassuringly, we found, once again, that most knockdowns of DV genes resulted in increased aggregate formation. We added the new results to the revised manuscript (Figure 5G-H in the manuscript, and main text: PP. 7; 19-20; 23).*

More detail is required for the cell lines and their driver mutations for HTT and CHD8.

*- To characterize the effects of CHD8 haploinsufficiency in human neurons, we designed a CRISPR-Cas9 single guide RNA (sgRNA) that targets near residue 1210. This sgRNA targets the same exon that harbors multiple de novo loss-of-function mutations. We co-transfected an EFS-Cas9-2A-EGFP plasmid and a U6-driven gRNA plasmid into HUES66 human embryonic stem cells (hESCs). After 4 days, cells expressing GFP were purified by fluorescence-activated cell sorting and collected for single cell-derived colony isolation. Using Sanger sequencing, we isolated two colonies with premature stop codon mutations (Q1248X, termed CHD8-A; G1247X, termed CHD8-B). The chosen guide was optimized to have minimal chance of off-target modification with the closest off-target site having three mismatches, including one in the critical seed region for Cas9 binding, which makes any off-target activity highly unlikely.*

*The HTT lines, both 180Q and 72Q isogenic systems, were previously described in detail in: An MC et al., Cell Stem Cell, 2012 and in Xu X et al., Stem Cell Reports, 2017, respectively. These are now added to the revised manuscript.*

In terms of statistics, the p values quoted for multiple t test results are below 10E-100, which is simply not plausible given the overlap in distributions e.g. in Figure 2B. More detail in methods, and a revision of the test results is required.

*- These statistics are correct. The actual statistics are 72Q:p=0.0051, 180Q:p=8.6057e-310, ASD: p=1e-323, and for mESC data: p=0. Instead of reporting these ridiculously small values, we reported those as <10^-10. The reason the p-values are so small is that they are based on a very large number of sample points, on the order of thousands of points from each sample, corresponding to all pairs of cells.*

Minor comments

The figure quality & resolution in the pdf is very poor. This is the case for all five figures.

*- The figure in the combined pdf file is in low quality. There is a possibility to download the original tiff files from the links in the pdf. We made sure that all figures are provided at high quality and high resolution.*

P-values are reported without naming tests, or inadequate description of test used, and whether assumptions have been met.

*- We thank the reviewer for catching this. There were two places where p-values are missing. In lines 259 and 339, following the reviewer’s comment, we added that the p-value related to a hypergeometric test. In lines 313 and 315, they continue the sentence from line 311, where it is stated that the test is Wilcoxon signed rank test.*

Ln 278-280 ‘This model also has computational advantages, as the NB model has no closed formula for the maximum-likelihood solution, which is even more difficult when a mixture-model parameters are to be deduced.’

That’s not a problem with the many computational approaches to estimating parameters via ML by using hybrid approaches with the EM, although, of course, less elegant to the EM presented by the authors in the supplementary methods..

*- We agree with the reviewer. There is nothing wrong with the hybrid approaches, and still they are less elegant to the EM we present in the manuscript. This is why we related to the advantages of our approach.*

Ln 285 “could be dominantly fitted by”. What do you mean by dominantly fitted? Best fit? How is this determined?

*- We refer to the Methods section at the end of the sentence, In the Methods section it is defined as a fit where the coefficient of the Gaussian part is larger than 0.9.*

Ln 341 “to reversely correlate” “to inversely correlate”?

*- Corrected.*

Ln 379 No such motif was found in the CHD8 model?

*- The motifs that were found for the CHD8 model did not have a p-value that was significant enough.*

Ln 410 why/how were the two control genes chosen? It would be good to see results for a few more control genes.

*- We chose eif2b2 as a representative of genes with similar mean expression but no variability change between WT and mutant HD cells. Nsa2 had a different average expression but was among the DV genes (i.e. , different variability rank) in the 180Q system, while the rest of the knockdown genes had similar expression with different variability. Following the reviewer’s comment, in the new NPC screen we performed we expanded both DV and control genes to include 15 genes each.*

Ln 411 how many constitute the “great majority” ? This is very imprecise. What statistical threshold was used?

*- In Figure 5E we explain how we define a significant increase in the aggregate formation in the knockdown samples compared to non-treated cells. Most of the samples are above this threshold.*

L436 Instead of using the phrase “investigating the time dimension” it would be more appropriate to say “investigate the age of onset and hence severity”

*- Following the reviewer’s comment we changed this phrase accordingly.*

L503 “titer”

*- Corrected.*

L509 “imbalance with three…”

*- Corrected.*

L570 “ snRNAseq” should be “scRNAseq”?

*- Corrected.*

L580 Please state whether paired end and read length, as well as targeted lib size.

*- Following the reviewer’s comment, we added these stats in the Methods section.*

L600 “Cells that had an N-E index smaller than 2 were discarded from further analysis.” Please demonstrate that this index was reasonable and robust to discriminate cells. Fig S3-F displays this ratio but it still seems a very arbitrary cut off with no clear separation.

*- The cutoff looks arbitrary because of the binning and the scale of the figure. Following the reviewer’s comment, we changed the binning and the scale such that it better demonstrates the separation (Figure R7).*

*Figure R7 (Figure S3F in the revised manuscript). The N-E ratio distribution.*

L641 Specify the total number of parameters for this model: 2 + 2 + 1 ? 2 from the weights/proportions of the three models, two for the normal/gaussian and one for the exponential.

*- The reviewer is correct. Following this comment, we added in the Methods section under “Mixture model for gene expression data” a sentence to clarify this point: “The total number of parameters in this mixture model is 5, including 2 for the proportions of the three distributions, two for the normal distribution parameters and one for the exponential.”*

L648 “ For the DVSM analysis, only genes with average expression larger than 5 normalized counts “ – It is not clear by what you mean here. Also, don’t you mean variation?

*- We agree with the reviewer that the sentence is not clear enough. We changed it accordingly: “For the DVSM analysis, only genes with average expression larger than 5 normalized counts and expressed in at least half of the cells in both conditions were taken into account.” -> “For the DVSM analysis, we included only genes that were expressed in at least half of the cells and had a large enough average expression level (> 5 normalized counts) in both conditions.”*

L655 Why is this a 3 parameter model? Are you assuming mu = 0, sigma = 1???

How good is the fit of these models? Please demonstrate through simulations and on your results – QQ plots etc. If the fitting is nonsense then the following analysis which makes use of the gaussian weights will be non-sense too.

*- The model is a 3-fit parameter model in the sense that it fits a combination of three distributions: It has 2 parameters for the relative contributions of the different distributions, and another 3 parameters: mu and sigma for the normal distribution, and lambda for the exponential distribution.*

*We demonstrate the accuracy of the fit in two ways. First, by showing the –log(MLE) for the fit of the different genes, and comparing them to other models (Figure S5A-B). Furthermore, we demonstrate the suitability of the fit by presenting several examples of gene expression distributions and their corresponding fits (Figure 3).*

F1 legend – p-value without test statistic.

*- Following the reviewer’s comment, we added the missing test statistics in Figure 1C.*

L763 define “GRN”

*- This is defined in the manuscript in line 244.*

L769 explain: “Error bars represent 1 std over 10 replicates (see Figure S4 and Methods).”

*- This is explained in detail at the beginning of the supplementary under the section “Gene correlation comparison”. In brief, due to biases that are introduced by different cell numbers from the different cell lines, we sub-sampled an even number of cells from both WT and mutant cells to perform the calculation. We repeated this procedure of sub-sampling ten times, and the std calculation is based on these. Following the reviewer’s comment, we referred to this explanation in the text: we changed “(see Figure S4 and Methods)” -> “(see Figure S4 and Supplemental Methods)”.*

FigS3G – There seem to be other cells that are also showing signals of being outliers very similar to the outlier that was removed. (at 15 & 55).

*- The reviewer is correct about cell #15, while cell #55 is within the inclusion criterion of 5 MADs. We marked only one cell with the arrow to attract the attention of the reader to the cell with the strongest outlier signature (i.e., smallest correlations to the rest of the cells). Following the reviewer’s comment, we added an arrow pointing at cell #15 as well (Figure R8).*

*Figure R8 (Figure S3G in the revised manuscript). Example of Spearman correlation between every 2 cells in the 72Q iPSC-derived NPCs. Arrows point at outliers.*

**Second round of review**

**Reviewer 1**

In the revised manuscript: "Pluripotent stem cell derived models of neurological diseases reveal early transcriptional heterogeneity" Sorek et. al report presents that transcriptional heterogeneity which presumably precedes neurodegeneration might be a feature of several distinct neurodegenerative (AD, HD) or even neuropsychiatric diseases (ASD).

The authors have provided a response to most of the raised concerns. Much of the response is new or edited writing and the new version of the manuscript has clearly been improved in my view and is more easily understandable.

Unfortunately, the most important criticisms have not been addressed and most important validation experiments have not been carried out. We are still concerned about the question of heterogeneity. This is relevant to both the quantified heterogeneity in the used datasets and the experimental validation.

1. The data analysis could be improved through inclusion of additional, publicly available datasets and validation in an independent cohort. I would be able to say that the analysis conducted is not convincing on its own but good enough if the experimental validation would be solid.

2. Still severe limitations in the experimental follow-up. Suggested experiments to use a well-defined cell type such as NGN2 neurons or sorted neurons etc. to decrease the experimental differences were not conducted. The characterization of the cells used are insufficient as criticized in the first submission. The connection between 1 and 2 vague.

For example, comparison between 180Q and 180Q corrected shows dramatic differences at d1-3 that will translate into dramatic differences during differentiation that is more likely to explain observed heterogeneity than the genetic difference.

The added knockdown experiments in NPCs are presented without error bars and the knockdown efficiency is so low for most targets. I am not sure how this can help the study. I suggest that the authors perform at least one well-controlled experiment to strengthen this important study. This could entail well-defined and relevant differentiated cells, sorted populations or additional control groups such as independent differentiated controls that don't show the observed transcriptional heterogeneity etc.

**Reviewer 2**

The authors have clarified several questions regarding modelling and statistical testing as well through performing additional simulations and adding further explanations which was welcome. The model fitting adequacy is not adequately addressed through merely considering maximisation of -ln L and several examples of where the model fits well. This should be assessed over all the genes.

The issue of somatic instability of the repeat was acknowledged but the manuscript not amended in the discussion to show this is a possibility (caveat)

The authors have now made all data available on GEO and are providing code on request for reproducibility. Can't this be put on GitHub?

**Authors Response**

**Point-by-point responses to the reviewers’ comments:**

Point-by-point rebuttal letter to Sorek et al., GBIO-D-20-00550R1

Reviewer #1: In the revised manuscript: "Pluripotent stem cell derived models of neurological diseases reveal early transcriptional heterogeneity" Sorek et. al report presents that transcriptional heterogeneity which presumably precedes neurodegeneration might be a feature of several distinct neurodegenerative (AD, HD) or even neuropsychiatric diseases (ASD). The authors have provided a response to most of the raised concerns. Much of the response is new or edited writing and the new version of the manuscript has clearly been improved in my view and is more easily understandable. Unfortunately, the most important criticisms have not been addressed and most important validation experiments have not been carried out. We are still concerned about the question of heterogeneity. This is relevant to both the quantified heterogeneity in the used datasets and the experimental validation.

1. The data analysis could be improved through inclusion of additional, publicly available datasets and validation in an independent cohort. I would be able to say that the analysis conducted is not convincing on its own but good enough if the experimental validation would be solid.

*During the revision process of our manuscript, a highly relevant single-cell RNA-seq dataset came out, comparing brains of Huntington’s disease (HD) patients to healthy brains (Lee H et al., Neuron, September 2020; PMID: 32681824), where the authors used a droplet-based method to evaluate the transcriptional profiles in adult HD (and control) brains. Analyzing these new datasets, we found a striking effect of increased heterogeneity in adult human HD (compared with healthy controls) in D1 and D2 medium spiny neurons (MSNs; New Figure 1d-e in revised manuscript), as well as in astrocytes (New Figure 1f in revised manuscript), strongly supporting our model. These new data have now been added to our revised manuscript as New Figure 1d-f and New Figure S1h-i. We fully trust that this new and important addition to the manuscript, together with our original validation experiments, is highly (and sufficiently) convincing.*

2. Still severe limitations in the experimental follow-up. Suggested experiments to use a well-defined cell type such as NGN2 neurons or sorted neurons etc. to decrease the experimental differences were not conducted. The characterization of the cells used are insufficient as criticized in the first submission. The connection between 1 and 2 vague.

*Comparing neurons with a neurodegenerative “phenotype” to control neurons is a notoriously difficult task. To overcome this issue, in the original manuscript we used HEK293 cells, which are frequently used in the polyQ field, where we were able to sort a homogeneous population with polyQ-aggregates and compared it to similar cells which did not form aggregates. This enabled us to test some of the targets we identified, and to validate our findings. However, following the Reviewer’s original comment, we repeated the HEK293 cells experiments using PSA-NCAM-sorted hPSC-derived neuronal progenitor cells (NPCs). Because polyQ aggregates are not normally formed in pluripotent cells nor in their neuronal derivatives (Victor MB et al., Nat Neurosci, 2018; PMID: 29403030), we had to perform several rounds of sorting until we were able to enrich for a polyQ-containing NPC population. Since we do not have polyQ-containing NGN2 inducible hPSCs, this experiment would entail, once again, repeated enrichments of polyQ populations following ectopic expression of polyQ-GFP (several months of work), but which would not provide a superior system over our existing neurons already derived by in vitro differentiation (and sorted for PSA-NCAM). But as mentioned in the answer to the first point above, the newly added scRNA-seq datasets of HD patients versus healthy brains is the most relevant experiment we could have hoped for, directly comparing transcriptional heterogeneity of HD to healthy brain cells. We believe that this newly added real patient data is more convincing than any cell culture experiment.*

For example, comparison between 180Q and 180Q corrected shows dramatic differences at d1-3 that will translate into dramatic differences during differentiation that is more likely to explain observed heterogeneity than the genetic difference.

*In our experience, the first few days of the 20 days differentiation protocol can vary between experiments, however by day 20 the cells acquire similar morphology. Moreover, at day 20 we maintained the cells for a few passages in order to increase the fraction of cells that differentiated into NPC and therefore establish a homogenous cell-state baseline. As a side note, there is growing evidence suggesting that long polyQ repeats may have early phenotypes, and hence the observed differences may indeed be real. Regardless, for all our experiments we do not rely on a single system and use several isogenic pairs.*

The added knockdown experiments in NPCs are presented without error bars and the knockdown efficiency is so low for most targets. I am not sure how this can help the study. I suggest that the authors perform at least one well-controlled experiment to strengthen this important study. This could entail well-defined and relevant differentiated cells, sorted populations or additional control groups such as independent differentiated controls that don't show the observed transcriptional heterogeneity etc.

*The added knockdown experiments in the manuscript are presented using a boxplot view and are based on three biological replicates. We believe that this is a much more straightforward view compared to only mean and standard deviation.*

Reviewer #2: The authors have clarified several questions regarding modelling and statistical testing as well through performing additional simulations and adding further explanations which was welcome. The model fitting adequacy is not adequately addressed through merely considering maximisation of -ln L and several examples of where the model fits well. This should be assessed over all the genes.

*Although we have graphically demonstrated the model fit for only several genes, we have assessed the log-likelihood over all genes in Figure S5A-B.*

The issue of somatic instability of the repeat was acknowledged but the manuscript not amended in the discussion to show this is a possibility (caveat).

*We have now added this to the discussion in the revised manuscript.*

The authors have now made all data available on GEO and are providing code on request for reproducibility. Can't this be put on GitHub?

*We have generated a public GitHub repository which contains all relevant main code at: Gitub:https://github.com/srmatan/neurological_early_transcriptional_heterogeneity.git*
